# Effects of spectral smearing on speech understanding and masking release in simulated bilateral cochlear implants

**DOI:** 10.1371/journal.pone.0287728

**Published:** 2023-11-02

**Authors:** Margaret Cychosz, Kevin Xu, Qian-Jie Fu

**Affiliations:** 1 Department of Linguistics, University of California, Los Angeles, Los Angeles, CA, United States of America; 2 Department of Head and Neck Surgery, David Geffen School of Medicine, University of California, Los Angeles, Los Angeles, CA, United States of America; University of Southern Mississippi, UNITED STATES

## Abstract

Differences in spectro-temporal degradation may explain some variability in cochlear implant users’ speech outcomes. The present study employs vocoder simulations on listeners with typical hearing to evaluate how differences in degree of channel interaction across ears affects spatial speech recognition. Speech recognition thresholds and spatial release from masking were measured in 16 normal-hearing subjects listening to simulated bilateral cochlear implants. 16-channel sine-vocoded speech simulated limited, broad, or mixed channel interaction, in dichotic and diotic target-masker conditions, across ears. Thresholds were highest with broad channel interaction in both ears but improved when interaction decreased in one ear and again in both ears. Masking release was apparent across conditions. Results from this simulation study on listeners with typical hearing show that channel interaction may impact speech recognition more than masking release, and may have implications for the effects of channel interaction on cochlear implant users’ speech recognition outcomes.

## Introduction

Cochlear implants (CIs) are widely successful prostheses that restore auditory sensations for individuals with severe-to-profound sensorineural hearing loss. However, despite advances in the implant’s electrical signal processing techniques, the speech signal that the CI conveys remains highly degraded. Spectral resolution in the CI signal is affected by the (1) number of activated electrodes [1], (2) proximity of healthy neurons to activated electrodes [2], and (3) current spread due to activation of multiple electrodes in the cochlea [3]. For the first factor, a greater number of activated electrodes theoretically translates to each electrode stimulating a smaller frequency region, permitting the user access to finer-grained spectral detail within the frequency spectrum. For the second factor, healthy neurons that are adjacent to dead regions targeted by implanted electrodes along the cochlea can be inadvertently stimulated. Both this phenomenon, as well as more general current spread from activated electrodes, distort the relationship between the frequency assigned to the electrode and what would be expected based on [4]’s frequency-to-place function.

Assuming ideal frequency allocation and tonotopic place of stimulation, all three of these factors reduce spectral resolution for adult and pediatric CI users, with ramifications for speech recognition [1, 5, 6], processing [7, 8], and categorization [9, 10], as well as music perception [11]. The poor spectral resolution in the CI signal likewise negatively impacts sound localization outcomes such as interaural time and level differences [12] (but see [13, 14]) and spatial release from masking (SRM) [15], including in adolescents with CIs [16].

The current work focuses on one aspect of reduced spectral resolution: channel interaction in the CI array stemming from spread at the electrode-neuron interface. Poor spectral resolution and increased channel interaction are correlated, and previous work has established that increased channel interaction results in poorer speech perception and recognition outcomes in both CI users and participants with normal-hearing (NH) listening to acoustic CI simulations [17–20]. In a CI, spectral cues, such as differing ratios of low- to mid-frequency energy concentrations to index vowel identity, are meant to stimulate spatially-distinct regions along the electrode array. Consequently, to identify relative or absolute spectral cues, the neural sites should be discrete and discriminable for users. However, when current spreads to adjacent electrodes in the array, stimulating multiple, overlapping sites, such spectral cues are clearly compromised—a well-known phenomenon of speech and music perception in CIs [8, 21]. More specifically, spectral smearing is known to collapse differences between peaks and valleys in the frequency spectrum, compromising important cues to vowel identity (formant peaks) and vowel/consonant nasality (spectral valleys, or anti-formants). Large amounts of spectral smearing shrink the acoustic vowel space, rendering vowels less distinguishable (in 4-channel vocoder simulations that manipulated the carrier band filter slope) [22]. And spectral smearing is one reason why CI users can have poorer pitch perception outcomes, since pitch is expressed through a single, relatively-low frequency [23].

An additional, complicating factor, however, could be differences in the degree of channel interaction *across* ears: minimal channel interaction in one ear might not guarantee successful speech outcomes for the CI user if the degree of interaction differs substantially interaurally in bilateral CI configurations. Interaural differences in other areas that characterize electrode array fit, such as tonotopic mismatch and insertion depth, inhibit binaural benefits for speech recognition and spatial release from masking in CI users [24] and subjects with NH listening to vocoder simulations [15, 25–27]. There is some suggestive evidence that, above and beyond uniformly increasing the degree of channel interaction in both ears, interaural differences in the degree of channel interaction worsen speech and sound localization outcomes in CI users and simulations [14]. First, interaural mismatch, in any form, is likely to impact SRM because listeners do not have consistent, reliable binaural cues to segregate incoming speech streams. However, the effects of interaural mismatch on SRM could be particularly difficult for across-ear differences in components such as frequency-place-mismatch and channel interaction because both of these components would compromise speaker identity cues (e.g. vocal tract length), making it more difficult to differentiate between talkers. Nevertheless, the true effects of interaural mismatches for the degree of spectral smearing are unclear, and they might vary by outcome (e.g., speech perception versus sound localization). Computing CI users’ functional spectral resolution interaurally, and evaluating its impact on users’ outcomes, is one approach to investigate this question. However, in CI users, functional spectral resolution stemming from *channel interaction* is, logically, often confounded with additional factors such as amount of residual hearing, auditory system health, and duration of deafness making it difficult to isolate effects of channel interaction upon users’ outcomes. In this case, a simulation study conducted on NH listeners, that tightly manipulates the amount of channel interaction while holding other variables constant, may help address the question.

This study presents listeners with NH with materials that are spectrally reduced in a manner similar to CI processing to evaluate how the degree of channel interaction within and across ears affects speech recognition thresholds (SRTs) and SRM. A group of subjects with NH listened to 16-channel sinewave vocoded speech simulating different degrees of channel interaction within and across ears. Target speech was delivered to both ears in one of two listening (masker) conditions: (1) diotic, with two maskers delivered simultaneously to both ears and (2) dichotic, with only one masker delivered to each ear for a total of two maskers. SRM was quantified as the difference between the average SRT in the diotic and dichotic listening conditions. However, note that typical SRM tasks (at least in acoustic hearing) are often administered differently, via the signal to noise ratio for a non-collocated versus collocated source.

## Methods

### Participants

Sixteen listeners with NH (8 males, 8 females) participated in this study (mean age = 26.38 years; range = 23–36). All participants were native speakers of American English and had some experience with vocoded speech from a previous vocoder study [15]. Participants had pure tone thresholds <20dB HL at all audiometric frequencies between 250–8000Hz, measured using a GSI61 clinical audiometer that is used regularly in the lab and annually calibrated. There were no inter-ear asymmetries. This study was approved by the Institutional Review Board at the University of California, Los Angeles and written, informed consent was obtained from all participants before commencing the study procedures.

### Conditions

The experiment consisted of three channel interaction conditions (broad channel interaction, limited channel interaction, mixed broad-limited interaction), each presented in the dichotic and diotic listening/masker conditions, for a total of six experimental conditions. The two maskers were presented simultaneously in both listening conditions. However, for the diotic condition the maskers were delivered simultaneously to both ears, and for the dichotic condition one masker was delivered to the right ear and the other masker to the left. The target sentence was always played diotically for participants.

The virtual simulation of spatial locations can be done through various techniques such as head-related transfer function (HRTF) filtering. However, In this study, HRTFs were not used. The dichotic condition in the present study was similar to the infinite interaural level difference (ILDinf) conditions from [28], in which the acoustic crosstalk from the contralateral interferer introduced by HRTF was artificially removed. [28] found that SRM was approximately 5dB or more for ILDinf than for HTRF-based processing, for both actual bilateral CI listening and for bilateral CI simulations. Similar differences were observed in our previous studies (1.8dB SRM in [29] with HRTF vs. 7.6dB SRM in [15] with dichotic listening). Therefore, the present approach allows for better stimulus control, the largest possible differences between the diotic and dichotic performance, and potentially larger SRM so that the effects of inter-aural mismatch in the degree of channel interaction on SRM can be better exploited.

### Materials

Target speech materials were 5-word sentences each composed of one of ten items from the following categories: Noun, Verb, Number, Color, Clothing (e.g., “John needs two green shoes.”). The first item in the target sentence was always the first name “John.” The sentences were always morpho-syntactically grammatical, but semantically neutral between items. The matrix-styled test paradigm used in this study employed a modified coordinate response test paradigm. Both these paradigms and matrix tests typically use closed-set paradigms. While a closed-set list does allow for more guesswork than open-set, we chose this stimuli set to build off of previous work manipulating additional CI processing parameters (e.g. frequency-to-place mismatch [15]). We acknowledge that some previous work on channel interaction has instead employed open-set lists [30, 31].

For each trial, the masker speech materials were likewise two distinct 5-word sentences, that also differed from the target sentence (e.g., “Bob finds two blue coats.”). All speech materials, target and two masker sentences, were produced by three distinct adult male speakers. The mean fundamental frequency (f0) across all 50 sentences for the target was 106 Hz. The mean f0s for the two masker sentences were 97 Hz and 128 Hz. The target and masker voices were consistent across trials. Stimuli were recorded at a 44.1kHz sampling rate with 16-bit quantification.

**Fig 1:**
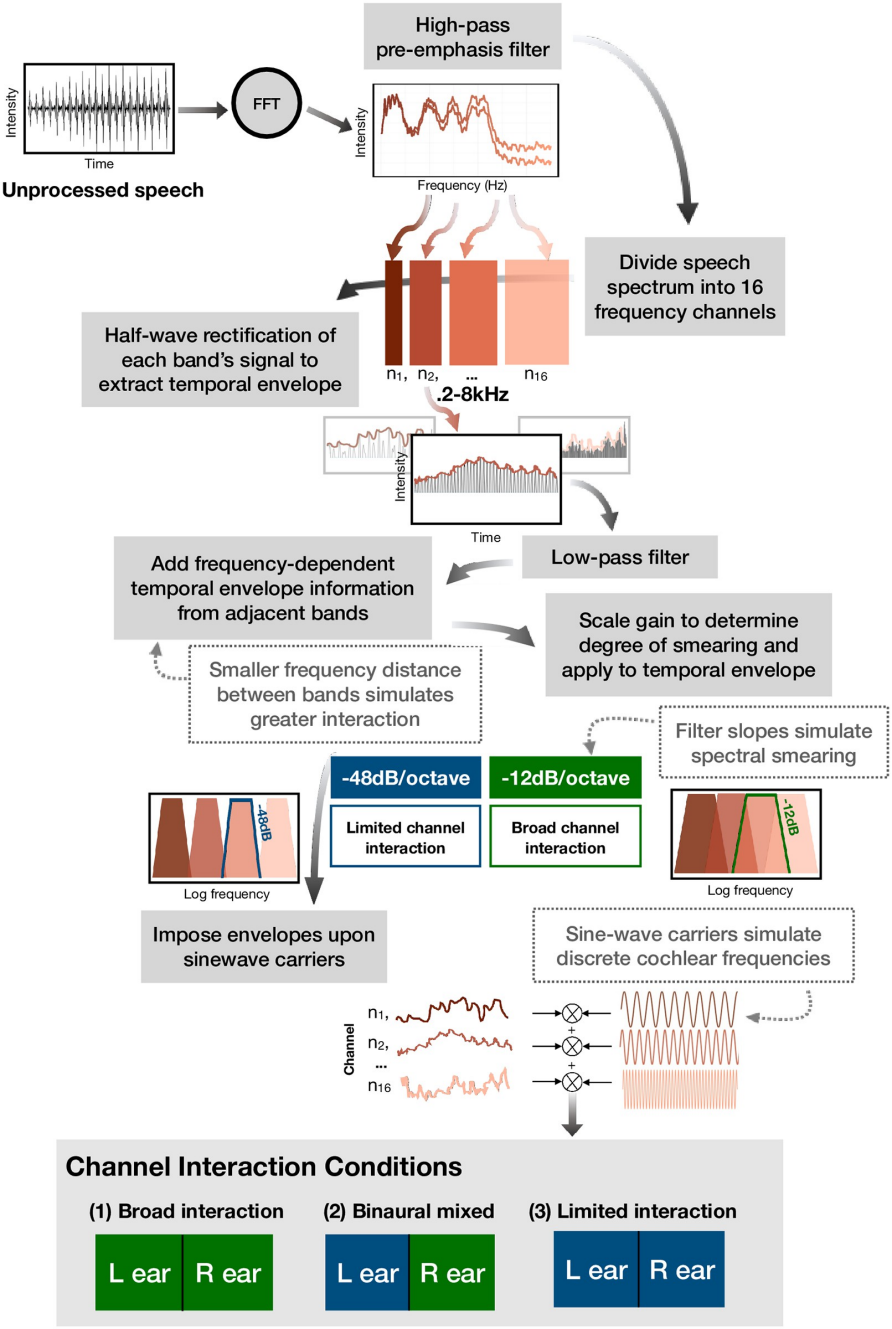
Signal processing workflow to generate the channel interaction experimental conditions. See text for further detail. 1. binaural broad (L12R12): Bilateral CI simulation with broad channel interaction within each ear but no inter-aural mismatch. 2. binaural limited (L48R48): Bilateral CI simulation with limited channel interaction within each ear and no inter-aural mismatch. 3. mixed-limited-broad (L48R12): Bilateral CI simulation with limited channel interaction within the left ear and broad channel interaction within the right ear.

All speech stimuli were processed to approximate an acoustic simulation of a CI using a 16-channel sinewave vocoder (Fig 1). First a high-pass, pre-emphasis filter (>1200Hz) with a -6dB/octave slope was applied to the original audio recordings (sampled at 44.1 kHz). The speech envelope (200–8000Hz) was divided into 16 different frequency bands (4th order Butterworth with a -24dB/octave slope). Within each band, the slow-varying temporal envelope was extracted using half-wave rectification and was low-pass filtered (<160Hz, the standard filter cut-off in vocoder research meant to emulate aspects of CI processing [32, 33]). Traditional vocoder approaches to simulating channel interaction employ noise carriers and vary the output synthesis filter slope (steeper slope simulating less interaction). The current procedure employed a sinewave carrier so channel interaction was simulated by adding variable amounts of temporal envelope information extracted across analysis bands to the temporal envelope of a particular band (see [34] for a similar implementation). In this way, the amount of adjacent envelope information depended both on the degree of channel interaction *and* the frequency distance between bands (greater amounts of interaction between bands that were closer in frequency). A gain factor was applied where the desired output filter slope could be specified (-48dB/octave for limited channel interaction or -12dB/octave for broad). Finally, the resulting temporal envelopes were used to modulate the amplitude of the sinewave carriers. Each sinewave carrier corresponded to the center frequency of its corresponding analysis band. Thus, the spectral information in the signal was reduced to an array of 16 modulated sinewaves simulating the implant electrode array. Three channel interaction conditions were generated for the experiment: binaural broad (L12R12), binaural limited (L48R48), and mixed-limited-broad (L48R12).

The CI simulations were vocoded using sinewave carriers, rather than noisebands. Although phoneme and sentence recognition performance has been shown to be similar with sinewave and noise-band CI simulations [35], sinewave simulations have been shown to better emulate real CI performance for pitch-related speech tasks [36], including intelligibility, speaker identification, and gender identification. This is especially true if the envelope includes frequencies up to at least 160 Hz [37, 38], which the current simulation does. Sinewave carriers also offer better stimulation site specificity and better temporal envelope representation than noise-band carriers. One of the main drawbacks of sinewave carriers—that they cannot emulate channel interaction—is mitigated by the signal processing approach that we take in this paper.

### Procedure

Participants completed the experiment in a sound booth, wearing headphones (Sennheiser HAD 200). Stereo stimuli were presented in real-time via audio interface (Edirol UA-25) connected to a mixer (Mackie 402). Target and masker speech were presented to participants at the desired signal-to-noise ratio (SNR) (see below for detail), which was defined as the root-mean-square (RMS) amplitude ratio of target speech to masker speech. Stimuli were sent via different channels and HRTFs were not applied. For the diotic listening conditions, the SNR was computed as the ratio between the target speech and the *total* energy of the two maskers presented in each channel (right or left). For the dichotic conditions, the SNR was the ratio between the target speech and the masker in each channel. The dichotic masker condition was similar to the “spatially separated” condition in [39] except that no HRTFs were used in this study. Masker RMS amplitude was always consistent across channels. With three listening conditions, and two masker conditions, there were 6 total conditions presented.

The SRT was computed in real-time using a 1-up/1-down adaptive procedure that aimed for participants to correctly recognize 50% of presented sentences [40]. Initial SNR was 10dB; it was manipulated over the total stimulus (target plus masker) and not manipulated separately for target and masker. A given trial proceeded as follows: the participant listened to the speech stimulus (target combined with maskers) and attempted to identify the *Number* and *Color* uttered. The participant would then choose among a closed-set list of 10 numbers and 10 colors presented on the screen. Correct identification of both number and color resulted in a decreased SNR (-4dB) for the subsequent trial while incorrect identification of either color or number resulted in an increased SNR (+4dB) for the subsequent trial. After two incorrect trials, the step was reduced to +2dB. Within each block, the SRTs were then computed from the average SNR of the last 6 steps (in the event of <6, another run was conducted).

Each participant completed at least two blocks (20 trials/block) for each of the 6 conditions. For each participant, and each condition, a single SRT was computed by averaging over all experimental blocks conducted. If the SRT differed by more than 3dB between block one and two, a third block was run (25/212 or 11.79% total blocks), and the average over the three blocks was the final SRT. To examine potential effects of block number on SRTs, we fit a linear model predicting the SRT computed from each condition and block. The parameter block number (factor variable: block 1, 2, or 3); block did not predict SRTs, and it did not interact with listening condition. Thus, there were no statistically reliable differences in SRTs, for any listening conditions, by block.

Participants did not complete any practice trials and no training or feedback were given. When subjects do not receive training or feedback, their recognition of vocoded speech improves very little [41]. Furthermore, training effects due to listening to the acoustic simulation were likely minimal given the short experiment duration (approximately 2 hours/participant), and we found no reliable differences in SRTs by block number (see previous paragraph), again suggesting minimal training effects. We also randomized the presentation of conditions across blocks, further delimiting potential learning effects.

## Results

We first report descriptive statistics and multilevel modeling on the SRTs in each listening condition followed by the SRM results. All results and modeling were conducted in the RStudio computing environment (v. 2022.07.0) using the lme4 and lmerTest packages for modeling, the emmeans package to evaluate and correct all pairwise comparisons, and ggplot2 for graphics [42–45]. Scripts to replicate these analyses are included in the OSF repository affiliated with this project (https://osf.io/23am9/?view_only=0a68dde7d8ba485fb46ea0841b758848).

### SRT results

**Fig 2:**
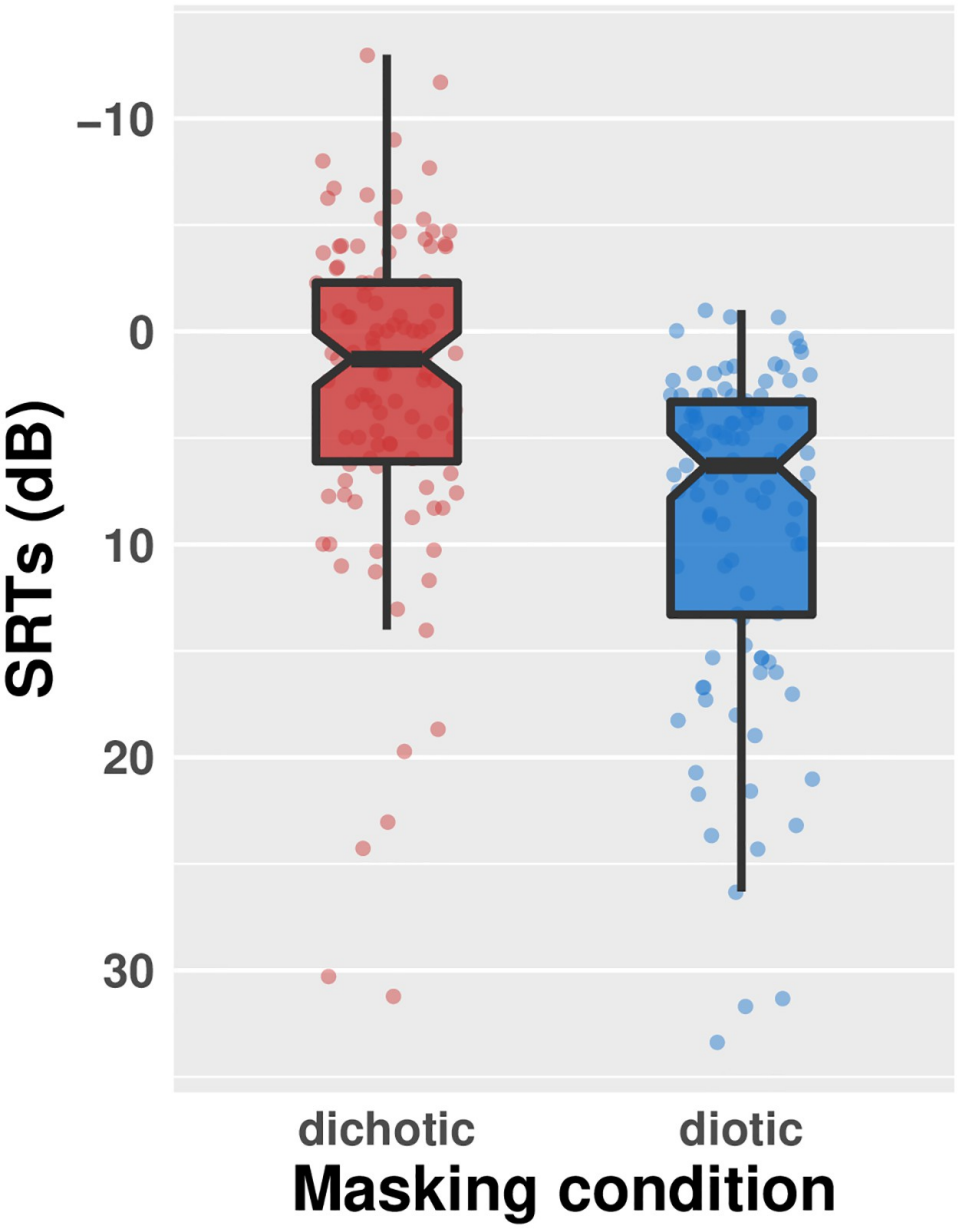
Speech recognition thresholds (SRTs) by listening condition. Smaller SRTs, at higher positions on the y-axis, indicate better recognition of test material (i.e. at more challenging signal to noise ratios). Colored points represent individual listener responses; points are horizontally jittered for readability. Boxes indicate interquartile range and whiskers 1.5 x the interquartile range in each direction. Black horizontal lines indicate the median and notches represent 95% confidence intervals surrounding the median.

**Fig 3:**
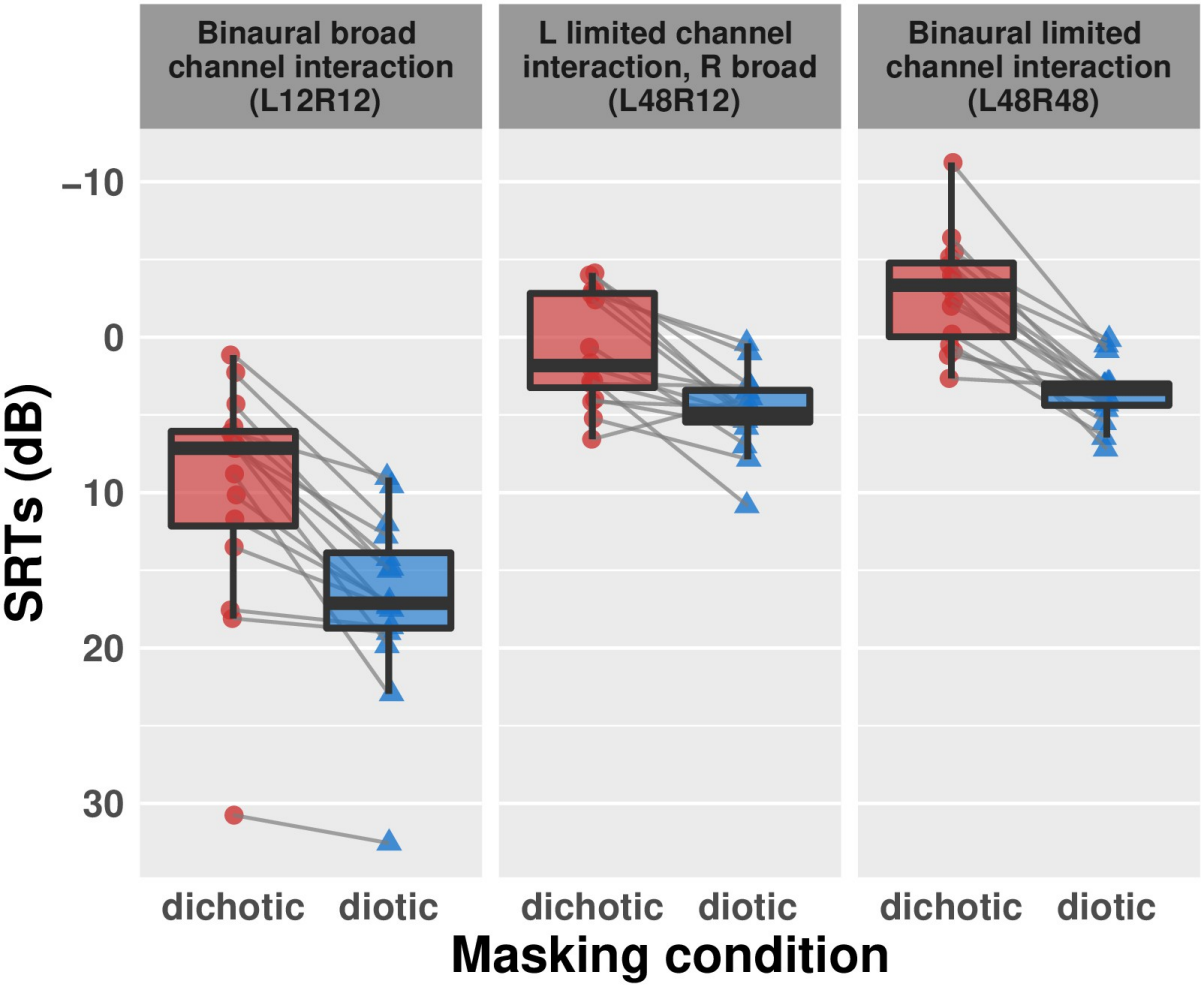
SRTs by channel interaction condition. Smaller SRTs, at higher positions on the y-axis, indicate better recognition of test material (i.e. at more challenging signal to noise ratios). Colored points represent individual listener responses; gray lines connect listener-level SRTs across masking conditions. Boxes indicate interquartile range and whiskers 1.5 x the interquartile range in each direction. Black horizontal lines indicate the median.

Unsurprisingly, SRTs were higher for diotic than dichotic masker presentation conditions (Table 1 and Fig 2). SRTs were also higher for the binaural broad channel interaction condition (L12R12) than the monaural broad-limited (L48R12) or binaural limited interaction conditions (L48R48) (Fig 3), suggesting a negative impact of increased channel interaction upon SRTs. The beneficial effect of the dichotic masker condition was consistent across the channel interaction conditions, but especially when the channel interaction was similar across ears (L12R12: 6.45 dB difference; L48R48: 6.51 dB).

**Table 1:** Descriptive statistics of SRTs (dB) by listening condition: Mean (SD) range.

Masker presentation	Combined channel interaction conditions	Binaural broad (L12R12)	L limited, R broad (L48R12)	Binaural limited (L48R48)
Dichotic	2.65(7.69)-13–31.2	9.84(7.76)0–31.2	0.92(4.03)-8–8.7	-2.96(3.88)-13–5.3
Diotic	8.8(7.6)-1–33.4	16.29(7.08)4.3–33.4	4.82(2.87)-0.7–11	3.55(2.16)-1–7.7

To complement description of these raw data, we additionally fit a linear mixed effects model to predict the effect of listening condition upon SRTs. The significance of potential model parameters was determined via comparing model log-likelihood values and AIC estimates. Categorical predictor variables were contrast-coded so the model intercept can be interpreted as the mean response across all conditions; positive model coefficients indicate higher SRTs. To account for individual variability and correlation stemming from participant-level effects, we included random intercepts by participant; all fixed effects were added step-wise to this baseline, random effect-only model.

Best model fit included the fixed effects of masker presentation (diotic vs. dichotic) and channel interaction (binaural broad vs. monaural limited and broad vs. binaural limited), but not their interaction (p>.05 for model comparison with and without interaction; see Table 2 for model summary). The lack of an interaction suggests that the benefit of dichotic > diotic is consistent in all three channel interaction conditions. Interpretation of model coefficients indicates a SRT increase of 5.7 dB in the diotic over dichotic conditions (t = 9.45, p<.001). The model likewise suggests a 2.64 dB greater SRT in the monaural limited-broad channel interaction over binaural limited channel interaction conditions (t = 3.52, p = .002) and a 10.23 dB greater SRT in the binaural broad channel interaction condition over the monaural limited-broad interaction condition (t = 14.01, p<.001). See model summary for full pairwise comparisons. The significant effect of masker presentation, without an interaction, indicates SRM in all channel interaction conditions which will be explored in the following section.

**Table 2:** Model predicting the effect of listening condition upon SRTs.

	Estimate	S.E.	t.value	p-value
Intercept	10.33	0.93	11.12	<.001
**Masker**				
Diotic	5.70	0.60	9.45	<.001
**Channel Interaction**				
L12R12–L48R12	10.23	0.73	14.01	<.001
L12R12–L48R48	12.87	0.74	17.47	<.001
L48R12–L48R48	2.64	0.75	3.52	.002

### SRM results

To further evaluate the effect of masker presentation, we evaluated a second outcome variable: SRM, or the difference between the average SRT in the diotic and dichotic masker conditions. Here, a higher value indicates greater release from masking or the listener benefiting more from the dichotic listening condition during the task.

**Fig 4:**
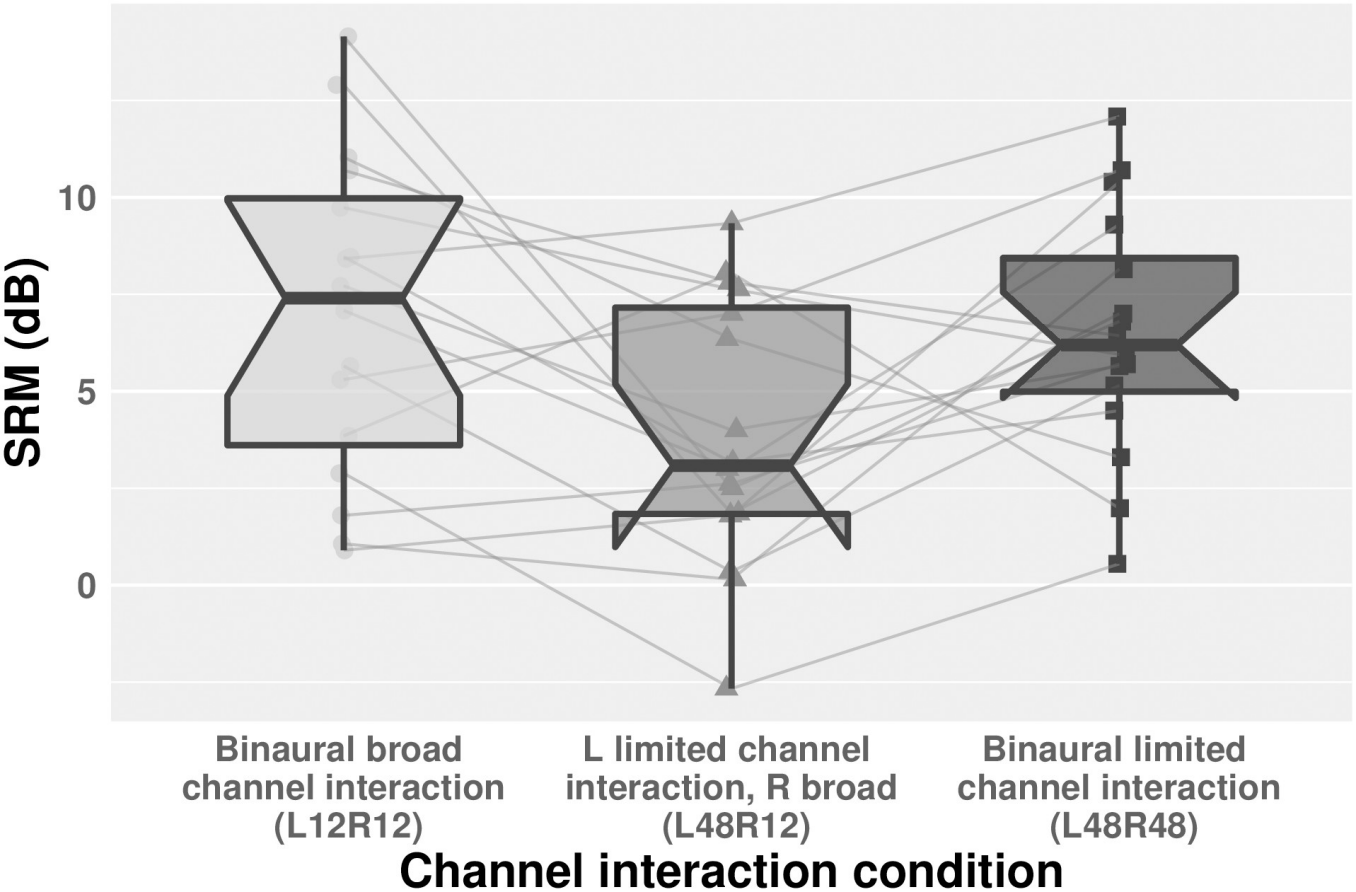
SRM (diotic-dichotic) by channel interaction condition. Higher values indicate greater benefit from dichotic over diotic listening conditions. Points represent individual listener responses; gray lines connect listener-level SRM values across channel interaction conditions. Boxes indicate interquartile range and whiskers 1.5 x the interquartile range in each direction. Black horizontal lines indicate the median and notches represent 95% confidence intervals surrounding the median.

Significant SRM was found for all channel interaction conditions. However, the SRM was greatest in the binaural broad and binaural limited channel interaction conditions (L12R12: M = 6.98 (SD = 4.16); L48R48: M = 6.48 (SD = 3.15), indicating greater SRM for same than different channel interaction (Fig 4).

To further evaluate these results, we fit another linear mixed effects model, again applying contrast coding to the categorical variables and controlling for participant-level effects in the random effect structure (random intercepts), to predict the effect of channel interaction condition upon SRM (see Table 3 for model summary and pairwise comparisons). Channel interaction condition improved upon a baseline, random effects-only model. Model coefficients show a 3.04 dB SRM difference for the binaural broad over the broad-limited condition, but just a 0.5 dB SRM difference for the binaural broad over binaural limited condition and -2.54 dB benefit for mixed channel interaction over limited interaction. Together, these results suggest that the most SRM occurs during *same* channel interaction conditions (broad or limited interaction), and that manipulating the degree of channel interaction affects SRM less than SRTs.

**Table 3:** Model predicting the effect of channel interaction condition upon SRM.

	Estimate	S.E.	t.value	p value
**Channel Interaction**				
L12R12—L48R12	3.04	0.71	4.31	<.001
L12R12—L48R48	0.50	0.71	0.71	0.759
L48R12—L48R48	-2.54	0.71	-3.60	0.002

## Discussion

This study examined the effects of spectral smearing (channel interaction) upon speech recognition and masking release by presenting sinewave vocoded speech to simulate the experience of listening through CIs. Results demonstrated (1) a negative effect of increased channel interaction upon speech recognition, (2) the presence of masking release despite the degree of channel interaction, and (3) greater masking release when the degree of channel interaction was minimal and similar interaurally. We elaborate upon these findings below.

### Effects of channel interaction

CIs users experience a reduced functional spectral resolution as a consequence of, most obviously, discrete electrode activation; however, poor spectral resolution is also caused by current spread between electrodes along the cochlea. As described in the introduction, the resultant spectral smearing—when channels peripheral to the target are inadvertently excited and adjacent electrodes become non-independent—inhibits CI users’ ability to segregate multi-channel information, such as speech [5].

The results corroborate a number of previous studies linking stronger channel interaction to poorer speech perception outcomes [18–20, 46]. However, this study goes a step further in demonstrating SRT improvements (-10.23 dB model estimate) in an interaural channel interaction mismatch condition, suggesting that reducing channel interaction in even one electrode array could improve speech recognition outcomes, despite a possible interaural mismatch in spectral resolution. Although in our preliminary work only three degrees of interaural channel interaction were tested (interaural broad, interaural limited, and interaural interaction mismatch), the fact that interaural mismatch did not interfere with speech recognition, instead improving it over the interaural broad condition, suggests that steps could be taken in the clinic to employ processing strategies that reduce channel interaction, even if such strategies cannot be optimally matched across ears or devices (see Study Limitations section for caveat on CI acoustic simulations). It is also important, however, to recall that there are a number of possible effects of *other* forms of interaural mismatch independent of spectral resolution, (e.g., insertion depth mismatch) that likewise impact SRM [15, 25–27].

There is some evidence that the relationship between channel interaction and speech perception outcomes is frequency- or even intensity-dependent (i.e., better performance when the place and intensity of stimulation match). Channel interaction may compromise lower-frequency acoustic cues (e.g., formant frequencies for vowels or formant transitions for consonants) more than higher-frequency cues (e.g., spectral energy peaks for voiceless fricatives). Indeed, among CI users, [19] only found a correlation between electrode discrimination (quantified as ability to distinguish between pulse trains presented to adjacent electrodes) and speech recognition for frequencies below 2680Hz, with the strongest correlation occurring for a frequency band spanning 1768–2650Hz. This latter range captures the second and third formant frequencies for adult male and female voices (indexing vowel frontedness and roundness, respectively); see also [20]. Consequently, an important subsequent step in this line of research will be to systematically manipulate the degree of channel interaction *across* the simulated electrode array, as well as how this interacts with interaural mismatch. A consistent spectral resolution across the array is not realistic, nor observed in CI users [20]. For example, there could be a negative impact of interaural channel interaction mismatch when (simulated) apical electrodes interact, but less so with basal. The current three-level channel interaction manipulation was not able to evaluate this aspect of interaural mismatch.

Masking release was found across all three channel interaction conditions, suggesting that channel interaction may impact speech recognition more than segregation outcomes. However, there were notable differences between the SRT and SRM outcomes. Unlike the speech recognition outcome, the least amount of SRM was instead found in the interaural mismatch condition. Listeners were able to employ the single channel of more resolved spectral information (less interaction) for SRTs, but not for SRM. Instead SRM may depend more on listeners being able to fuse two input signals into a single auditory image; so a more degraded signal, assuming interaural consistency, may be preferred over finer spectral resolution in just one ear. In this way, our simulation work corroborates [47] who examined SRM in a population typically characterized by poor binaural processing outcomes: bilateral CI users with asymmetric hearing (typically longer periods of deafness in one ear). In that study, many CI users experienced SRM upon hearing the target in the *better*-performing ear, but not the poorer, again demonstrating the importance of being able to fuse two monaural signals.

### Study limitations

Vocoder simulations attempt to emulate the experience of listening via one or more CIs. Acoustic CI models permit researchers explicit control over parameters such as channel interaction or insertion depth that are confounded within individual CI users and typically not under experimental control. Simulations also allow the study of CI parameters without changing a CI user’s speech processing strategy. Reprogramming strategies can be a less-than-ideal experimental manipulation since CI users often require some time to adapt to new strategies and users’ listening experiences could be adversely affected.

Nevertheless, vocoder simulations do not mimic the experience of using a CI. Acoustic models often diverge from CI users’ actual behavior [48]. And unlike CI users, the participants with NH listening to vocoder simulations have not had months or years of experience processing electrical speech signals and creating frequency-to-electrode maps [49], including binaural differences in such maps [24]. Furthermore, while CI users have been shown to adapt to severe changes in processing strategies (frequency allocation) within just three months [50], and NH listeners can similarly cope with frequency re-mapping on shorter timescales with explicit instruction [51], the participants with NH in this study clearly did not have such opportunities. Consequently, like all studies employing vocoding to study CIs, this study cannot estimate CI users’ quantitative patterns or declare how speech recognition or masking release would unfold as a function of spectral smearing among CI users; rather, results should be interpreted as performance trends.

Besides signal vocoding, another important limitation is the channel interaction simulations themselves. In this study, only three distinct interaction simulations were tested (broad, limited, and mixed broad-limited), in two masking conditions. Going forward, it would especially be helpful to understand how the *degree* of interaural channel interaction mismatch impacts speech recognition and masking release. For example, interaurally matched limited and broad channel interaction improved SRM over interaural mixed limited-broad (+3.04 and +2.54 dB model estimates, respectively). It is somewhat surprising that interaural channel interaction mismatch would worsen SRM above and beyond broad channel interaction in both ears—the former at least presents listeners with a single channel of more fine-grained spectral information. Instead, the results suggest that while channel interaction alone may impact SRTs, interaural mismatch may be more detrimental for SRM, an idea that further manipulations in the degree of interaural channel interaction could explore.

A final limitation of this study is the outcome measures evaluated. SRTs and SRM are important, yet relatively coarse, metrics to evaluate speech perception outcomes in CIs. A low SRT cannot indicate how speech and language would actually be processed under certain conditions of spectral degradation. For example, a low SRT does not imply robust lexical processing or index how users perceive different acoustic contrasts, such as spectral (e.g. /s/—/∫/) versus temporal (e.g., /t/—/d/), that are known to be differentially degraded through CIs. So future work on this topic could examine potential effects of signal degradation, such as interaural mismatch in channel interaction, upon specific speech perception or processing outcomes [17].

### Clinical implications

CI users tend to have the strongest speech outcomes when using processing strategies with which they have the most experience, suggesting that users can overcome characteristics of signal degradation, such as spectral smearing and poor frequency resolution, with time and listening experience. Nevertheless, even if users can adapt to degradation over the long term, reducing the degree of channel interaction in the short-term or during initial fittings may be worthwhile and has been shown to improve users’ speech perception outcomes [52]. These results corroborate this idea, and the lower SRTs for interaural mismatches suggest that speech processing strategies that prioritize reductions in channel interaction even in one ear may improve speech recognition outcomes overall. Consequently, the focus on reducing channel interaction wherever possible, as opposed to matching interaction interaurally, is an important translational outcome of this work. However, since the effect of interaural mismatch did vary by outcome—minimal interaural differences benefited listeners’ SRM more than SRTs—it is clear that more fine-grained experimental manipulations of interaural channel interaction mismatches are needed to determine best clinical practices.

During implant programming, there are two methods to reduce channel interaction: current focusing and electrode deactivation [52]. Electrode deactivation does indeed improve CI users’ speech understanding outcomes [53]. However, the manipulation comes at a cost—namely reduction in spectral resolution since there are fewer sites, and thus fewer neurons, stimulated along the cochlea. Focused stimulation, on the other hand, has the benefit of tightening the electrode-to-neuron interface selectively without necessarily sacrificing spectral resolution. (There are, nevertheless, limitations to the clinical usefulness of focused stimulation related to overall power consumption and inadvertent channel spreading generated by high stimulation rates [54]).

These results might suggest that clinicians should prioritize minimizing channel interaction, even at the expense of interaural mismatch. However, it is impossible to directly translate results from CI acoustic simulations and direct work with CI users. For example, [52] selectively de-activated high-threshold (i.e., broad channel interaction) electrodes in N = 8 adult CI users (9 ears). The authors did not find that processing programs that prioritized minimal channel interaction significantly improved CI users’ speech perception outcomes. Consequently, before further clinical recommendations can be made concerning prioritizing minimizing channel interaction versus interaural mismatch, further work is need on binaural CI users—which is what was simulated in the current study—to evaluate if reductions in channel interaction improve speech perception outcomes in that population.

## Supporting information

S1 Appendix (PDF)
