# Supplementary material for: Effects of spectral smearing on speech understanding and masking release in simulated bilateral cochlear implants
Source: PLoS One. 2023 Nov 2;18(11):e0287728. doi: 10.1371/journal.pone.0287728 (PMC10621938; doi:10.1371/journal.pone.0287728)
Supplement: S1 Appendix — (PDF) [file pone.0287728.s001.pdf]

1 Appendix

Table 4. Detailed parameters for the 16-channel sinewave vocoder, including the corner frequencies of each analysis and carrier band as well as the center frequency of the carrier sinewave for each analysis and carrier band.

	**Input**			**Output**		
Channel	Low	High	Center frequency	Low	High	Center frequency
1	200	275	235	200	275	235
2	275	367	318	275	367	318
3	367	479	419	367	479	419
4	479	616	543	479	616	543
5	616	782	694	616	782	694
6	782	985	878	782	985	878
7	985	1231	1101	985	1231	1101
8	1231	1532	1373	1231	1532	1373
9	1532	1899	1706	1532	1899	1706
10	1899	2345	2110	1899	2345	2110
11	2345	2889	2603	2345	2889	2603
12	2889	3551	3203	2889	3551	3203
13	3551	4358	3934	3551	4358	3934
14	4358	5342	4825	4358	5342	4825
15	5342	6540	5911	5342	6540	5911
16	6540	8000	7233	6540	8000	7233
